# Rhythm, movement, and autism: using rhythmic rehabilitation research as a model for autism

**DOI:** 10.3389/fnint.2013.00019

**Published:** 2013-03-28

**Authors:** Michelle W. Hardy, A. Blythe LaGasse

**Affiliations:** Center for Biomedical Research in Music, Colorado State UniversityFort Collins, CO, USA

**Keywords:** autism spectrum disorders, synchronization, movement regulation, neurologic music therapy, rhythm

## Abstract

Recently, there has been increased focus on movement and sensory abnormalities in autism spectrum disorders (ASD). This has come from research demonstrating cortical and cerebellar differences in autism, with suggestion of early cerebellar dysfunction. As evidence for an extended profile of ASD grows, there are vast implications for treatment and therapy for individuals with autism. Persons with autism are often provided behavioral or cognitive strategies for navigating their environment; however, these strategies do not consider differences in motor functioning. One accommodation that has not yet been explored in the literature is the use of auditory rhythmic cueing to improve motor functioning in ASD. The purpose of this paper is to illustrate the potential impact of auditory rhythmic cueing for motor functioning in persons with ASD. To this effect, we review research on rhythm in motor rehabilitation, draw parallels to motor dysfunction in ASD, and propose a rationale for how rhythmic input can improve sensorimotor functioning, thereby allowing individuals with autism to demonstrate their full cognitive, behavioral, social, and communicative potential.

## Introduction

Autism Spectrum Disorder (ASD) is traditionally characterized by deficits in social interaction, communication, and restricted repetitive and stereotyped patterns of behaviors, interests, and activities (American Psychiatric Association, 2000). Although there will be changes to the diagnostic criteria with the DSM-V, the proposed criteria maintain focus on social/communicative symptoms and restricted/repetitive behaviors (Wing et al., 2011; Nishawala, 2012). This focus is maintained despite research demonstrating an extended profile of ASD that includes movement impairments (Staples and Reid, 2010; MacNeil and Mostofsky, 2012; Whyatt and Craig, 2012). Movement research has vast implications for treatment and therapy for individuals with autism, as the coordination and regulation of sensory and movement information is required for social interaction, speech communication, and participation in the environment (Donnellan et al., 2012). Since movement is a new area of consideration in ASD, there are few research studies focused on accommodations or treatments to improve movement in children with ASD. One accommodation that has not yet been explored is the use of rhythmic cueing to improve sensorimotor functioning in ASD.

Recent comprehensive research reviews have established that auditory rhythmic cueing is an effective tool for gross motor rehabilitation in populations including stroke (Bradt et al., 2010) and Parkinson's disease (de Dreu et al., 2012). Success observed with these populations has been attributed to processing of rhythm in the brain, motor synchronization to an auditory stimulus, and the intact motor synchronization ability of persons with neurological disease and disorder. Furthermore, rhythmic cueing is proposed to activate motor neurons via reticulospinal pathways, effectively priming the motor system (Rossignol and Melvill Jones, 1976; Thaut, 2005). Based on this rehabilitation research, we propose that rhythm may also be used in the treatment of movement differences in individuals with ASD. The purpose of this paper is to illustrate the potential impact of rhythmic cueing for sensorimotor regulation in persons with ASD. To this effect, we review research on rhythm in motor rehabilitation, draw parallels to motor dysfunction in autism resulting primarily from differences in the cerebellum, and propose a rationale for how rhythmic input can improve motor functioning, based on initial research indicating that rhythmic synchronization is intact despite cerebellar deficits. Furthermore, the potential benefits of rhythm for social/communicative behaviors will be explored.

## Rhythm in rehabilitation

Over the past two decades, researchers have begun to understand the neurological basis of music in the brain. Researchers have demonstrated that music processing and production are distributed throughout the cortex, subcortex, and cerebellum (Peretz and Zatorre, 2005). Areas engaged with music perception and production are not unique to music; rather, they overlap with non-musical networks (Thaut, 2005; Patel, 2011). Furthermore, the distributed nature of music in the brain allows preservation of musical functions despite the loss of a related non-musical function. For example, in persons with non-fluent aphasia, the ability to sing is unimpaired despite loss of speech production (Özdemir et al., 2006; Schlaug et al., 2009b). Research findings in music neuroscience have led to the development of neurologic music therapy techniques that drive cortical plasticity for rehabilitative gain.

Elements of music have been used effectively in therapy in collaboration as well as in isolation for various therapeutic needs. These elements include melody, harmony, tempo, dynamics, timbre, form, and rhythm. The organizing factor in all music is rhythm; therefore, rhythm serves as a timekeeper in the therapeutic application of music for motor rehabilitation goals and is foundational to auditory-motor synchronization. Auditory rhythmic cueing refers to an auditory sound stimulus with a fixed inter stimulus interval (such as the output from a metronome). As rhythmic cueing is well-documented to facilitate motor improvements in persons with neurological impairments, this paper will hypothesize the role of auditory rhythmic cueing in motor improvement in autism.

Bengtsson et al. (2009) demonstrated that auditory rhythm activates motor areas of the brain including the pre-motor cortex, supplementary motor areas, pre-supplementary motor area, and the lateral cerebellum. Rhythm not only activates motor areas of the brain, there is evidence of rapid motor synchronization to an external rhythmic cue in persons with and without neurological disability (Thaut et al., 1999a). Initial evidence of auditory-motor synchronization led to investigation of the auditory-motor pathway, with suggested involvement of the reticulospinal connections (Rossignol and Melvill Jones, 1976), cerebellum, brainstem, and the basal ganglia (Thaut and Abiru, 2010). This unique relationship between rhythm and motor function has been studied extensively in the rehabilitation sciences, where auditory rhythmic cueing has been utilized as an effective treatment in motor rehabilitation for over a decade (Thaut, 2005). There are two primary factors that contribute to the success of auditory rhythm in rehabilitation (1) rhythmic synchronization and (2) evidence that rhythmic cueing used systematically can facilitate cortical plasticity.

### Rhythmic synchronization

Rhythm refers to the division of time through distinguishable order and patterns of events, objects, symbols, or signs. It is one of the most important organizational aspects of music (Thaut et al., 1999a). Rhythmicity plays a critical part in learning, development, and performance, as timing of movement is essential in many motor control and cognitive functions (Thaut et al., 1999a, 2009; Molinari et al., 2005). Rhythm formation can integrate basic levels of sensory perception and motor entrainment into complex cognitive processes and motor adaptations (Thaut et al., 1999a). Findings in rhythmicity and brain research provide evidence that interaction between auditory rhythm and motor responses can be effectively employed for rehabilitation in movement disorders (Thaut et al., 1999a).

The role of rhythm in movement rehabilitation has been well-established through studies focused on persons with neurological disease and disorder. Thaut and colleagues found that rhythmic auditory cueing facilitated immediate improvement in gait parameters of persons with neurological injury, specifically cadence, velocity, and stride length (McIntosh et al., 1997; Thaut et al., 1997, 2001; Hurt et al., 1998). They concluded that the motor system is physiologically very sensitive to arousal by the auditory system and that rhythm facilitated positive change in motor output. Rhythm not only affected the timing of movement, but the total movement pattern. Specific findings indicate that auditory rhythmic cues add stability in motor control immediately (within two or three stimuli) rather than through a gradual learning process (Kenyon and Thaut, 2000). Rhythmic entrainment of neural auditory and motor impulses is based on motor synchronization to auditory signal frequency. This suggests that rhythm provides time information across the duration of the movement and not just the endpoints of movement when a response is matched to the period of the rhythmic signal.

The concept of frequency entrainment or period matching can happen at subliminal levels of sensory perception and allows “the brain to map and scale smoother time parameters of position change” due to the precise reference interval (Thaut et al., 1999a, p. 105). This research provides evidence of auditory-motor coupling, where the auditory external cue acts as a “forcing function” that optimizes the efficiency of kinematic movement parameters (Thaut et al., 1999a). Several studies have demonstrated that rhythmic synchronization is an effective tool for rehabilitation of gait in persons with Parkinson's disease (Miller et al., 1996; Thaut et al., 1996; McIntosh et al., 1997; Prassas et al., 1997; Howe et al., 2003; Arias and Cudeiro, 2008; Rochester et al., 2009), traumatic brain injury (Hurt et al., 1998; Kenyon and Thaut, 2000), spinal cord injury (de l'Etoile, 2008), stroke (Thaut et al., 1997; Roerdink et al., 2007, 2009; Hayden et al., 2009), Huntington's disease (Thaut et al., 1999b), and in patients with cerebellar ataxia (Abiru et al., 2008). Results of one study indicated that gait gains with 6 weeks of rhythmic auditory stimulation lasted 4 weeks after cessation of the treatment intervention in persons with Parkinson's disease (Kadivar et al., 2011). Researchers have also demonstrated success with rhythmic cueing for upper body rehabilitation goals for arm hemiparesis. Results of upper limb studies indicated decreased movement variability, increased speed of movement, smoothing of the movement trajectory (Thaut et al., 2002), and a decrease of compensatory trunk movements that often accompany arm movements in persons with stroke (Malcolm et al., 2009). These results are due to the neurobiological basis of timing in motor control and the impact of auditory-motor cueing.

The profound impact of rhythm on the motor system strongly suggests that the vital element linking music to motor behavior is time. Although the exact neurobiological process of rhythmic synchronization remains unclear; researchers have demonstrated that different aspects of processing time information are attributed to the neocortex, basal ganglia, cerebellum (Thaut et al., 2009) and thalamus (Krause et al., 2010). Regarding the cerebellum, researchers have shown that the cerebellar cortex and vermis contribute to the production of a timed motor response, particularly if it is complex and/or novel (Penhune et al., 1998; Thaut et al., 2009). From these analyses, the cerebellum makes a specific contribution to movement timing, aiding in computing the temporal parameters of incoming sensory stimuli and outgoing movements as well as in novel, temporally precise motor responses (Penhune et al., 1998; Thaut et al., 2008). Other data using auditory rhythmic cues evidenced contrasting cerebellar activation patterns associated with motor vs. perceptual and cognitive functions and that activation was neuroanatomically distinct based on function (Thaut et al., 2009). Because tasks of daily living require sensory input for successful completion, the cerebellum is likely necessary for the successful timing of these functions and the complex exchange between sensory and motor brain circuits (Molinari et al., 2007).

Individuals with cerebellar impairment show deficits in motor planning (Fisher et al., 2006) and adaptation (Block and Bastian, 2012); however, are relatively unimpaired in sensory adaptation (Block and Bastian, 2012). An intact sensory adaptation may be utilized to impact motor networks. Molinari et al. (2005) found that patients with cerebellar impairment demonstrated unimpaired motor synchronization to an external auditory cue. These findings suggest a direct drive between auditory and motor structures that may be pertinent for rehabilitation strategies in movement disorders (Molinari et al., 2005). Intact synchronization ability also demonstrates that even in the presence of cerebellar damage, temporal information could be available for the motor system through the auditory system to elicit functional change (Molinari et al., 2007).

The tight relationship between auditory rhythmic stimuli and motor responses has been demonstrated across many studies (Malcolm et al., 2008; Thaut et al., 2008, 2009). The evidence demonstrates that auditory-motor synchronization occurs rapidly and is maintained with tempo changes below conscious perception (Kenyon and Thaut, 2000; Tecchio et al., 2000). This subconscious adaptation is maintained in patients with cerebellar damage (Molinari et al., 2005), suggesting that the distributed nature of rhythm in the brain and underlying networks can function despite damage. This has further been demonstrated in gait rehabilitation of a person with cerebellar ataxia (Abiru et al., 2008). Observation of intact synchronization ability in persons with cerebellar differences suggests that rhythm could be utilized with other disabilities that show indication of cerebellar differences, including ASD.

### Rhythm and cortical plasticity

Researchers have demonstrated cortical changes in persons who engage in music over time. Compared to non-musicians, adult musicians have differences in auditory areas, sensorimotor areas, and areas involved in multisensory integration (Gaser and Schlaug, 2003; Bermudez and Zatorre, 2005; Imfeld et al., 2009; Luo et al., 2012). Since it is expected that a life-long musician would have differences in the brain, researchers have sought to determine if short-term training could change neural connections. Hyde et al. (2009) demonstrated that 15 months of musical training changed motor, auditory, frontal, and occipital regions in the brains of children with no formal musical training. Schlaug et al. (2009a) demonstrated changes in the anterior corpus callosum after 29 months of instrument training. Evidence of cortical plasticity has also been demonstrated in adults. Pascual-Leone (2001) showed increased connectivity of the hand area of the sensorimotor cortex after only a few weeks of training on the piano. Furthermore, musical training has been shown to increase responses of motor and pre-motor areas of the brain.

Listening to rhythmic sequences engages cortical areas for movement, even in the absence of the movement or planning to complete the movement (Bangert et al., 2006; Bengtsson et al., 2009). Furthermore, Lahav et al. (2007) demonstrated that non-musicians who received a short period of musical training increased activations in the motor areas of the brain when listening to the same music. Cortical plasticity has also been documented in persons with neurological disability following rhythmic interventions. Luft et al. (2004) and Whitall et al. (2011) demonstrated greater activation of motor areas including the pre-central gyrus, supplementary motor area (Whitall et al., 2011), and the cerebellum (Luft et al., 2004) following a 6-week bilateral arm training intervention paired with auditory rhythmic cueing. This intervention was compared to a dose-matched intervention without rhythmic cueing. This research demonstrates that short periods of engagement with rhythmic cueing can drive cortical plasticity, promoting structural and functional connectivity changes in the brain.

The above research demonstrates that auditory-motor coupling is not only possible, but occurs in persons who have neurological disease and disability. Although auditory rhythms have been applied widely in rehabilitation sciences, rhythm for synchronization has not yet been used to improve motor outcomes in individuals with ASD. Current neuroscience and motor research suggests that children with ASD have motor differences, which may benefit from the application of rhythmic cueing.

## Motor control in autism spectrum disorders

Motor disturbances are not part of proposed DSM-V diagnostic criteria of autism; however, growing evidence indicates that neurological dysfunction may be associated with abnormal movements seen in individuals with autism. In a study of 67 children with ASD, Hilton et al. (2012) found that 83% of children with ASD presented motor scores at least one standard deviation below the general population, with greater impairments seen in children with more severe autism. This was compared to 6% of children from the same families without ASD showing a similar deficit. Other studies have estimated that between 80 and 90% of children with ASD demonstrate some degree of motor abnormality (Ghaziuddin and Butler, 1998; Dziuk et al., 2007; Ming et al., 2007; David et al., 2009). Although studies focused on incidence of motor abnormality are relatively recent, the earliest description of ASD included motor differences.

In Kanner's earliest paper describing autism (1943), he acknowledged a variety of motor differences in the individuals with autism he observed. He noticed that while all of the children demonstrated skilled fine muscle coordination, several of the children exhibited clumsiness in gait and gross motor performances. He observed individuals with autism failing to assume an anticipatory posture as well as passive positioning like “a sack of flour” (p. 243). In some cases, the individuals he studied lacked the ability to adjust their body to the person holding them. Kanner also noted that the children's verbal utterances and motor performances were monotonous and repetitive, “resulting in marked limitation in the variety of spontaneous activity” (p. 245). He proposed that if the slightest element of a given action was modified, altered, or removed, it was not considered the same or accepted as such by the child (Kanner, 1943).

Since these earliest reports, researchers documented motor impairments in ASD including clumsiness and poor coordination (Hallett et al., 1993; Teitelbaum et al., 1998; Vernazza-Martin et al., 2005), gait abnormalities (Hallett et al., 1993; Vernazza-Martin et al., 2005; Rinehart et al., 2006), impaired performance of skilled motor tasks (Dewey, 1995; Mostofsky et al., 2006; Dziuk et al., 2007), and motor planning deficits (Rinehart et al., 2001; Schmitz et al., 2003; Gidley Larson et al., 2008; Fabbri-Destro et al., 2009). Motor deficits could have vast implications for communicative and social functioning, as these skills rely on the organization of sensory and motor responses (Donnellan et al., 2012). Teitelbaum et al. (1998, 2002) indicated that motor differences were apparent in infancy, including differences in the development of infantile reflexes, which may be indicative of a neurological difference in early childhood. Although researchers have documented abnormalities in nearly every brain system in persons with ASD (Minshew, 1994; Ciaranello and Ciaranello, 1995; Courchesne and Allen, 1997), MRI, and autopsy studies have consistently reported that the cerebellum is a common site of neuroanatomic abnormality.

Differences observed in the cerebellum of persons with ASD have included hyperplasia or hypoplasia of cerebellar hemispheres (Murakami et al., 1989; Hardan et al., 2001; Pierce and Courchesne, 2001) and one or more regions of cerebellar vermis (Pierce and Courchesne, 2001; Schmidt, 2002; Allen and Courchesne, 2003), and differences in the presence of Purkinje neurons (Kemper and Bauman, 1998; Allen and Courchesne, 2003). Fatemi et al. (2012) recently reviewed literature on the cerebellum in ASD and found points of consensus including abnormal anatomy, abnormal neurotransmitter systems, deficits in cerebellar motor and cognition, and neuroinflammation. The authors of this consensus paper presented evidence of a clear difference in cerebellar anatomy and function; however, the exact implications of these differences and treatment options are not yet understood (Fatemi et al., 2012).

Allen and Courchesne (1998) suggested that, based on connectivity, the cerebellum must function in both a general and highly integrative manner. These researchers suggested that the cerebellum may be responsible for purposes within multiple domains inclusive of cognitive, sensory, affective, and motor functions (Allen and Courchesne, 2003). Due to this widespread connectivity, Schmahmann and Pandya (2008) proposed that the cerebellum is involved with automatizing and optimizing functions around a “homeostatic baseline”; indicating that the cerebellum coordinates cognitive and emotional functions in the same way that it regulates and controls motor activity. Just as the cerebellum predicts the neural systems needed for a particular motor action, researchers suggest that it also predicts neural systems needed for a motor operation and then prepares for the operation at hand (Akshoomoff et al., 1997; Courchesne and Allen, 1997; Allen and Courchesne, 1998).

If the fundamental role of the cerebellum were to predict the neural systems needed to plan, adjust, and execute movements, then cerebellar damage or disease would likely affect the optimal functioning of a given neural system (Allen et al., 2004). Furthermore, the cerebellum's role in planning and coordinating movement would result in a lack of coordination in a person with cerebellar damage (Holmes, 1939). Persons with cerebellar damage maintain the ability to move; however, the quality and efficiency of movements is altered (Robinson, 1995). Although other neural systems continue to perform, they will do so sub-optimally without the preparatory role of the cerebellum to aid in performance (Allen et al., 2004). It is clear that individuals with ASD have the ability to move; however, Allen et al. (2004) proposed that they lack coordination due to a preparatory deficit. This preparatory deficit may account for the overall intact gross motor system that manifests differences in coordination and motor planning.

## Can auditory rhythm enhance motor regulation in ASD

The findings that rhythmic synchronization behavior is intact in patients with both atrophic and focal cerebellar lesions suggests the possibility that rhythm could be utilized in motor differences in ASD despite the presence of cerebellar abnormalities. One of the findings in the cerebellum in autism is abnormal development of vermal lobules VI and VII (Carper and Courchesne, 2000). These areas are known to process auditory information on a more arousal-oriented level and activation of lobule VI revealed a time sensitive response to modifications of the rhythmic tempo (Thaut et al., 2009). In rhythmic synchronization tasks, Stephan et al. (2002) showed different cerebellar circuit involvement in varying aspects of the presented tasks. Activation in vermal regions ipsilateral to the movement was consistent in novel and/or complex tasks and resulted in activation that was incrementally larger in these areas (Stephan et al., 2002; Molinari et al., 2007). Rhythmic auditory cues may therefore facilitate activations in these areas to elicit shared networks for motor performance, or if needed, may provide compensatory accommodations to activate other areas. Further research in this area is needed in order to assess the impact of rhythmic auditory-motor cues in therapy in this population.

One proposed function of the cerebellum as “comparator” is to adjust motor output related to planned actions (Penhune et al., 1998; Zatorre et al., 2007). The cerebellum predicts the timing of an upcoming movement, utilizes sensory feedback from the current movement, compares ongoing performance to an internal model, and then adapts responses such as force and/or trajectory (Penhune et al., 1998; Zatorre et al., 2007). Schmitz et al. (2003) observed that children with ASD exhibited latency of movement events, indicating over reliance on proprioceptive feedback to maintain postural stability. Bower (1996) suggested that the posterior cerebellar vermis coordinates proprioceptive input from muscle stretch receptors to optimize motor control. If there are cerebellar differences in ASD, the integration and response to feedback may be challenged or require additional accommodation. Since auditory feedback has been utilized to aid in proprioceptive muscular control (Thaut et al., 1999a; González and Yu, 2009; González et al., 2010), rhythmic auditory stimuli may create a feed-forward interaction directly influencing motor output in a predictive way (Zatorre et al., 2007). This would provide the person with ASD an accommodation facilitating more efficient and fluid movement without over-reliance on proprioceptive feedback.

The application of rhythm may serve to facilitate sensorimotor synchronization in autism, but based on other implicated deficits, it may contribute not only to gross motor functioning, but also related perceptual motor responses. One such perceptual motor skill is anticipatory preparation of movement. Gerloff et al. (1998) demonstrated that internal pacing of movement poses higher demands on the motor system than external pacing. Since individuals with autism exhibit deficits in anticipation (Rinehart et al., 2001) and appear to lack sufficient internal cueing, an external auditory rhythmic cue could provide a temporal template for organization of motor output while simultaneously decreasing the demand and increasing efficiency of movement. Auditory rhythmic stimuli can serve as predictable timing cues that influence motor anticipation resulting in the response pattern gradually becoming automatized (Thaut et al., 1999a).

Adams and Chambers (1962) and Schmidt (1968) investigated the automatization process of motor patterns and suggested that anticipation appeared to be “variable depending on the predictability of external response cues,” and temporal and spatial predictability of those cues was most influential on anticipation. Because rhythmic tracking requires predictability of the stimulus, typical participants demonstrated anticipation as their motor responses were slightly ahead of the beat [as cited in Thaut et al. (1999a)]. Patients with cerebellar impairment showed intact rhythmic synchronization and evidenced similar anticipatory responses (Molinari et al., 2007); therefore, an auditory-motor stimulus of rhythm would likely impact anticipatory preparation in movement in persons with autism. Treatment geared toward building an anticipatory means of motor control in autism might then facilitate the development of internal models for motor planning.

Motor planning or praxis is an essential skill for motor function. Studies have shown that persons with autism exhibit a generalized praxis deficit (Mostofsky et al., 2006; Dewey et al., 2007; Dziuk et al., 2007) and that this may be attributed to perceptual motor elements (Vanvuchelen et al., 2007). It has been found that children with autism are not able to translate their motor intention into a global action, but that they program single acts independently from each other (Fabbri-Destro et al., 2009). External cueing of rhythm might lessen the internal demands placed on an individual with autism by providing precise reference intervals at each stage of the movement. This might allow for an increase in fluency and accuracy of movement parameters, as well as organization of the overall movement sequence.

Although rhythm has not yet been studied in this context with persons who have ASD, the above evidence suggests that motor differences observed including planning and coordination of movement might benefit from auditory rhythmic cueing. It is important to note that this is not simply listening to rhythms; rather, interventions involve the application of rhythm at a tempo appropriate for facilitating a movement pattern or increasing motor stability. The development of interventions using rhythm for motor skills may also include other musical elements such as pitch, structure, and dynamics. These elements can further emphasize motor patterns and engage the client in the therapeutic process. Clinical music therapy interventions often involve music with a strong or embedded rhythm. The emphasis is on the temporal aspect; however, melody, lyrics, structure, and style are incorporated for motivation.

### Neurologic music therapy

Neurologic music therapy is the therapeutic application of music to cognitive, sensory, and motor dysfunctions due to neurologic disease or disability. Neurologic music therapy is a particular method of music therapy that was developed from neuroscience models of music perception and production (Thaut, 2005). Treatment in neurologic music therapy is focused on the use of rhythm and music stimuli to drive cortical plasticity. Traditionally, music therapy has been utilized to address social, communicative, and cognitive needs of children with ASD (e.g., Kern and Humpal, 2012). There are no systematic studies investigating the use of neurologic music therapy for movement disturbances in autism, likely due to the focus of social and communication skills in the diagnostic criteria. However, based on the above findings of movement differences in autism and the use of rhythm for other gross motor deficits, rhythm may be an appropriate accommodation for motor skill acquisition in ASD. Evidence that rhythm can be used for motor gains in a person with cerebellar ataxia can be used as a theoretical basis for using rhythm and music for movement in ASD.

Thaut (1988) derived a clinical motor rehabilitation model based on auditory-motor research. Within this model, auditory rhythmic signals as external stimuli can facilitate temporal muscular control of movement patterns by: (1) influencing timing and potentiation of motor neuron discharge, (2) decreasing muscular fatigue sensation, (3) facilitating automatized movement performance through the temporal predictability of its timing cues, (4) improving reaction time and response quality through facilitated response anticipation, and (5) providing auditory feedback for proprioceptive control mechanisms (p. 130). These elements have more recently been translated into neurologic music therapy techniques to address range of motion, muscle strength and endurance, muscle control and coordination, motor planning, and functional motor skills (Thaut, 2005). For example, music instrument playing has been utilized to improve functional reach in persons who have had a stroke (Sutton, 1984; Neugebauer et al., 2008; Altenmüller et al., 2009). Given motor deficits due to neurological differences in ASD, it is reasonable to apply these techniques to persons with ASD to address motor skills. Furthermore, the use of music may be especially effective due to unique responses to music in the brain.

There have been several reports that persons with ASD have enhanced pitch and/or melodic perception (Bonnel et al., 2003, 2010; Ouimet et al., 2012; Stanutz et al., 2012) Recently, Lai et al. (2012) demonstrated that low functioning children with ASD had stronger activations of the inferior frontal gyrus and superior temporal gyrus in song than in speech, exceeding cortical responses of typical children in the song condition. Furthermore, a greater activation of frontal-posterior networks was observed within the song condition, suggesting that children with ASD may be more effectively engaged in musical stimuli. Similar studies investigating rhythmic processing have not yet been conducted and research on perceptual timing in ASD is inconclusive (Falter et al., 2012). However, if music processing is enhanced or similar to that seen in typical children, the overall evidence provides a basis for using music embedded with a strong auditory rhythmic cue for greater global awareness within a task. Neurologic music therapy interventions would use rhythm as an accommodation, providing a temporal template for the completion of complex motor tasks that require chaining of motor acts through activation of compensatory neural networks.

The predictability of musical stimuli and the use of stimuli to improve motor planning may have additional effects on cognitive, communicative, and social functioning. Although the research presented thus far has been focused on sensorimotor deficits, evaluations of movement might serve as effective comparisons for deficits in parallel systems important for socialization and communication (Mostofsky et al., 2007). This may be observed in the timing involved in speech production or the ability to predict and respond to social cues in the moment. Motor regulation is required for postural control, gesture, facial expression, speech production, social interaction; all processes that are documented as impaired in ASD (Robledo et al., 2012). There may even be cases where motor deficit or difficulty appears as behavior; for example, the individual throwing an object or falling on the floor. In some cases, the individual may understand the necessary action, but fail to complete the action due to motor planning deficits. Therefore, improvements in motor functioning due to predictability and anticipation of rhythmic and musical stimuli may facilitate or improve functioning in other areas including social and communication skills.

The impact of auditory rhythms utilized to promote functional skills in therapy may also benefit other skill areas, due to the highly predicable nature of rhythmic stimuli. One study demonstrated that musical stimuli with a strong rhythmic foundation increased synchronized alpha and gamma bandwidths, as measured by Electroencephalogram, which corresponded to improved memory (Thaut et al., 2005). If systematically applied rhythmic stimuli can increase the timing of networks beyond movement, interventions may yield greater impact. This may be one reason that studies on music therapy and ASD have demonstrated improved social (Brownell, 2002; Kern and Aldridge, 2006; Kim et al., 2008; Finnigan and Starr, 2010) and communication skills (Lim, 2010; Wan et al., 2011). Changes documented in these studies may even be in part due to improved ability to regulate motor patterns and interact with the environment. Improvement in motor functioning would allow individuals with autism to demonstrate their full cognitive, social, and communicative potential as demands in these domains typically require a movement to respond such as a gesture or initiation.

## Conclusion

Autism is primarily defined with social and communication deficits; however, current literature suggests that movement differences play a part in autism and that this component warrants further investigation. If clinical treatment of autism addressed motor deficits, appropriate therapeutic goals to impact functional change might include motor coordination, motor planning, and functional motor skill development. Rhythmic auditory cueing could be an appropriate technique to provide a predictable structure to stabilize variability in the movement pattern and facilitate a motor plan. Given the current evidence, this is an area where further research is required to better understand the potential impact of rhythm on movement in persons with ASD.

### Conflict of interest statement

The authors declare that the research was conducted in the absence of any commercial or financial relationships that could be construed as a potential conflict of interest.
